# West Nile Virus Induced Cell Death in the Central Nervous System

**DOI:** 10.3390/pathogens8040215

**Published:** 2019-11-01

**Authors:** Bi-Hung Peng, Tian Wang

**Affiliations:** 1 Department of Neuroscience, Cell Biology and Anatomy, University of Texas Medical Branch, Galveston, TX 77555, USA; bpeng@utmb.edu; 2 Department of Microbiology & Immunology, University of Texas Medical Branch, Galveston, TX 77555, USA; 3 Department of Pathology, University of Texas Medical Branch, Galveston, TX 77555, USA; 4 Sealy Institute for Vaccine Sciences, University of Texas Medical Branch, Galveston, TX 77555, USA

**Keywords:** West Nile virus, neuronal death, central nervous system, apoptosis

## Abstract

West Nile virus (WNV), a mosquito-borne, single-stranded flavivirus, has caused annual outbreaks of viral encephalitis in the United States since 1999. The virus induces acute infection with a clinical spectrum ranging from a mild flu-like febrile symptom to more severe neuroinvasive conditions, including meningitis, encephalitis, acute flaccid paralysis, and death. Some WNV convalescent patients also developed long-term neurological sequelae. Neither the treatment of WNV infection nor an approved vaccine is currently available for humans. Neuronal death in the central nervous system (CNS) is a hallmark of WNV-induced meningitis and encephalitis. However, the underlying mechanisms of WNV-induced neuronal damage are not well understood. In this review, we discuss current findings from studies of WNV infection in vitro in the CNS resident cells and the in vivo animal models, and provide insights into WNV-induced neuropathogenesis.

## 1. Introduction

West Nile virus (WNV), is a member of the family of Flaviviridae, genus Flavivirus, a group of plus-sense, single-stranded RNA viruses. WNV was originally isolated in 1937 from the blood of a febrile woman in the West Nile region of Uganda and was introduced into the United States (US) in the summer of 1999. During 1999–2018, more than 50,000 cases of human disease were reported to the Centers for Disease Control and Prevention (CDC), including about 24,000 cases of neuroinvasive disease and 2300 deaths [1]. The clinical spectrum of acute WNV infection in humans ranges from a flu-like febrile condition to more severe neuroinvasive conditions, including meningitis, encephalitis, acute flaccid paralysis, and death [2]. In addition, up to 50% WNV convalescent patients have been reported to develop long-term neurological sequelae [3,4,5,6]. Currently, there is no specific therapeutic agent for treatment of the infection. No approved vaccine is available for humans. Neuronal death is a hallmark of WNV-mediated meningitis and encephalitis. However, the underlying mechanisms of WNV-induced neuronal damage are not well understood. Here, we summarize recent findings from studies of WNV infection in vitro in the central nervous system (CNS) resident cells and the in vivo animal models, and provide insights into WNV-induced neuropathogenesis.

## 2. WNV Induces CNS Cell Death Preferentially in the Neurons and Predominantly via the Apoptotic Pathway

WNV can get access to the CNS by multiple possible mechanisms, including the direct infection of the vascular endothelium [7], crossing the blood brain barrier (BBB) following its compromise [8,9], carried by transport immune infected cells via a “Trojan horse” mechanism [10,11], infection of the olfactory neurons [12,13], and direct axonal retrograde transport [14,15,16]. The main CNS residential cells, including neurons, and glial cells are presumably the target cells for WNV [17,18]. Both cell types have been shown to be permissive to in vitro WNV infection [19,20,21]. Analysis of the autopsied neural tissues of WNV encephalomyelitis patients also revealed viral infection in both neurons and glia cells [22]. WNV infection in mouse primary neurons triggers rapid replication kinetics and subsequently leads to cell death. In comparison, infections in non-neuronal glial cells are slowly progressive. For example, WNV infection in astrocytes was shown to evolve slowly and continued to produce infectious virus for more than three months [23]. Although WNV does not affect glial cell viability, it induces the secretion of inflammatory cytokines, such as tumor necrosis factor (TNF)-α, or neurotoxic factors, which together contribute to neuronal cell death [22]. Furthermore, the production of interleukin (IL-)1 in WNV-infected astrocytes during the convalescent stage of WNV infection decreases neurogenesis and ultimately leads to memory dysfunction [24].

**Figure 1:**
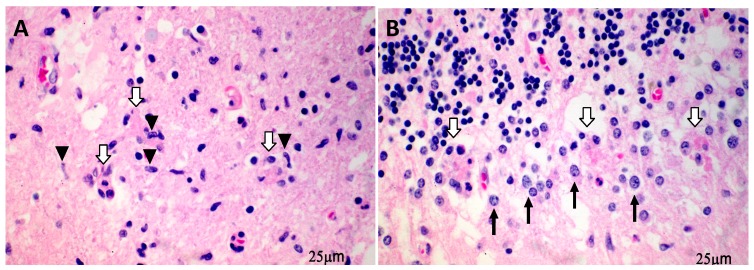
**Neuronophagia in a fatal human WNV case.** Two histologic sections of a fatal human WNV infection case are shown. Perivascular lymphocytic cuffing’s and neuronophagia (destruction of neurons by microglial cells), were the two main features under the microscope. Motor neurons in the lower region of the spinal cord were not present. In the upper cervical cord dying neurons (**A**, hollow arrows) were encircled and phagocytized by inflammatory cells, mainly microglia (arrowheads) as an example of neuronophagia. It was also found in Purkinje cells (**B**, hollow arrows) in the cerebellum and associated inflammation (Burgmann gliosis, arrows) in the surrounding area.

WNV can induce various types of programmed cell death pathways depending on the viral dosage. A high dose of WNV infection in Vero cells usually leads to necrosis. In contrast, a low dose of WNV triggers predominantly apoptosis in neurons, which is initiated by the release of cytochrome c from the mitochondria and formation of apoptosomes, and followed by the activation of caspase-9 and -3 then cleavage of the poly (ADP-ribose) polymerase [25]. Parquet M et al. [26] was the first to report apoptotic features in WNV-infected neurons, including cell shrinkage, chromatin condensation, and subdiploid DNA content [26]. Autophagy, another type of programmed cell death pathway, is known to support viral growth following infection with other flaviviruses, such as dengue virus or Japanese encephalitis virus [27,28]. Autophagy was also detected in WNV-infected mouse primary neuron culture and in ex vivo organotypic brain slice cultures [29]. However, Vandergaast R et al. [30] reported that the virus did not induce autophagy in human cell lines derived from brain and other tissue origins. Importantly, there are possible other neuronal death pathways involved in WNV infection in the CNS either by itself or assisted by other CNS cell types. Microarray analysis of brain tissues of WNV-infected young mice revealed the upregulation of genes associated with pyroptosis and necroptosis [31]. However, more studies are needed to validate the involvement of these cell death events in the CNS and their possible roles in exacerbation of the outcome of CNS diseases. In a human case of fetal WNV infection [32], WNV antigen and viral RNA were detected in the postmortem brain tissues and the spinal cord. A few months after the initial symptoms in the lower extremities, motor neurons were not present in the lower level of the spinal cord in the patient. Interestingly, we also noted in this patient neuronophagia, a destruction of neurons by microglial cells in the upper cervical cord indicating of a progressive inflammatory process. As shown in Figure 1A, dying neurons were encircled and phagocytized by inflammatory cells, mainly microglia, which is an example of neuronophagia. This was also noted in Purkinje cells (Figure 1B) in the cerebellum with astrogliosis in the surrounding area. In addition to the spinal cord and cerebellum, the brainstem and hippocampus (pyramidal neurons) were the other two main regions where neuronophagia was found.

## 3. Mechanisms of Neuronal Death Induced by WNV Infection

WNV infection in the CNS induces apoptosis preferentially in neurons. Results from the in vitro and in vivo animal models of WNV CNS infection suggest that both host and viral factors are involved in mediating virus-induced programmed cell death (Table 1)

### 3.1. Viral Proteins

#### 3.1.1. WNV Capsid Protein

It has been shown that WNV induces apoptosis in embryonic stem (ES) cell-derived neurons within 48 h post infection [20]. These results suggest that WNV can directly trigger neuronal death in the absence of inflammatory cell types, such as T cells, microglia, and monocytes. Yang et al. [33] first demonstrated that transfection of WNV capsid protein in neuronal and other non-neuronal cells led to apoptosis. They also found that mice injected stereotactically with a plasmid expressing WNV capsid protein exhibited neuronal death 1–2 days post injection, as evidenced by the TUNEL assay. In vitro cell culture studies further suggest that WNV capsid protein induces apoptosis by disruption of the mitochondrial transmembrane and activation of the caspase-9 and caspase-3 pathways. The 3′- terminal region of the capsid protein is associated with its apoptosis-inducing function [25,33]. Furthermore, it was reported that the phosphorylation of WNV capsid protein by protein kinase C enhanced its binding and nucleus co-localization with the HDM2 protein, which then blocked the formation of the HDM2 and p53 complex, thereby causing the stabilization of p53 and the subsequent induction of its target apoptotic protein, Bax [34,35]. The expression of the WNV capsid protein in non-neuronal glial cells by a Sindbis virus-derived vector (SINrep5-WNVc) also contributes to induction of neuronal death by triggering the release of neurotoxic factors, the induction of proinflammatory genes, and the suppression of OASIS (old astrocyte specifically induced substance). This was also evidenced in a rat model, in which striatal implantation of SINrep5-WNV(C) induced neuroinflammation and CXCL10 upregulation, and diminished OASIS expression [22]. Despite the substantial evidence supporting the pro-apoptotic effects of the capsid protein, a more recent report showed that expression of the protein increases the levels of phosphorylated Akt, a prosurvival kinase that blocks Fas-induced apoptosis. The discrepancy between these reports is likely due to the inclusion of an 18-amino-acid-residue signal peptide of prM in the capsid protein in the earlier reports [36].

#### 3.1.2. WNV Nonstructural (NS) Proteins 

The NS proteins of WNV also contribute to the induction of apoptosis. The expression of NS proteins in neuronal cells was shown to trigger the proapoptotic cyclic AMP response element- binding transcription factor homologous protein (CHOP)-dependent cell death [37]. There were elevated levels of TUNEL-positive Neuro-2a cells following transfection with a WNV NS2B– NS3 expressing plasmid, and NS3 alone, but not NS2B, triggered cell apoptosis. NS3 expression was linked to an apoptotic pathway mediated either individually or together by the caspase-3 and caspase-8 pathways. Both the protease and helicase domains of WNV NS3 protein are critical for apoptotic induction [38]. The NS2A protein can also facilitate viral cytopathicity and virulence in the absence of IFN-α/β response and the amino acid at position 30 of the NS2A protein is essential for performing this function [39]. WNV replicons and WNVs harboring a subset of either NS2A or NS3 mutations were reported to decrease the cytopathic effect in cell culture [40].

### 3.2. Host Factors Contribute to Neuron Death

#### 3.2.1. Caspase Pathways and Endoplasmic Reticulum (ER) Stress Responses

WNV infection disrupts the mitochondrial transmembrane and the activation of the caspase-9 and caspase-3 pathways in primary cortical neurons [25]. It was reported that WNV infection following treatment with caspase-3 inhibitors or in caspase-3^-/-^ mice led to a significantly decreased neuronal death in various areas in the brain, such as cerebral cortices, brain stems, and cerebella [41]. These results further support that the caspase-3 is a key positive regulator of WNV-induced apoptosis in neurons.

WNV also induces the upregulation of ER stress genes in neurons, including GADD153, BiP, and PKR-like ER kinase (PEK) expression. In comparison, the induction levels of these genes in WNV-infected astrocytes are variable [22]. The unfolded protein response (UPR) is an ER-mediated response to the accumulation of large amounts of unfolded or misfolded proteins in the ER. UPR can trigger cell death after severe or prolonged ER stress. One early report [37] showed that WNV triggers three major UPR pathways, inositol-requiring enzyme 1-dependent splicing of X box binding protein 1 (XBP1) mRNA, activation of activating transcription factor 6 (ATF6), and protein kinase R-like ER kinase-dependent eukaryotic initiation factor 2α (eIF2α) phosphorylation in human neuroblastoma cells and primary rat hippocampal neurons. Among them, ATF6 is important for maintaining cell viability and modulating immune responses upon WNV infection. Indeed, ATF6-deficient cells had higher cell death rates due to the abhorrent UPR signaling [42]. eIF2α is also associated with induction of the proapoptotic cyclic AMP response element-binding transcription factor homologous protein (CHOP), which promotes apoptotic cell death. The UPR also enhances the ER-assisted degradation (ERAD) of misfolded proteins. Ma et al. performed genome-wide screening using the CRISPR-Cas9 system in human 293 T cells and identified seven genes associated with WNV-induced cell death. These seven genes are all involved in the ERAD pathway, including EMC2, EMC3, SEL1, DERL2, UBE2G2, UBE2J1, and HRD1. The association of these genes with WNV-induced apoptosis was also confirmed in Neuro-2a cells [43].

#### 3.2.2. Proinflammatory Cytokines, Interferon Stimulating Genes (ISGs), and the Underlying Pathogen Recognition Receptor (PRR) Pathways

The induction of higher levels of proinflammatory cytokines, including IL-1β, -6, -8, and TNF-α, is associated with an increase in neuronal death following in vitro WNV infection in human neuroblastoma cells (SK-N-SH cells) as well as in the brains of WNV-infected Type 2 diabetic mice [44,45]. Multiple innate signaling pathways are involved in mediating CNS cell death via the regulation of inflammatory cytokine responses. For example, Sterile alpha and HEAT/Armadillo motif (SARM) is a highly conserved Toll/interleukin-1 receptor (TIR)-containing adaptor protein. It is preferentially expressed in the CNS tissues. SARM can restrict brain injury by modulation of TNF- α and microglial activation in mice [46]. The NLRP3 inflammasome pathway and IL-1β signaling also play key roles in controlling WNV infection in the CNS by restriction of its replication in neurons [47]. Osteopontin (OPN) is a negative regulator of a caspase 1-mediated, caspase 8- independent pathway that increases caspase-3 and inflammasome components. OPN was reported to prevent apoptosis in the CNS by critically controlling inflammation. As a consequence, OPN knockout mice demonstrated rapid induction of acute innate immune cytokines following WNV infection, which led to the subsequent inflammatory and apoptotic responses [48]. There was conflicting evidence on the proapoptotic effect of inflammation. For example, TNF-α expression was shown to reduce neuronal death by interactions with its receptor TNFR1, an effect that further down- regulates neuronal CXCR3 expression, diminishes its ligand CXCL10-mediated calcium transients, and ultimately delays caspase-3 activation [49].

ISGs also play a dual role in induction of neuronal death. For example, Ifit1 expression is anti- apoptotic as deficiency of Ifit1 was associated with increased neuronal death in vivo, which was both cell-intrinsic and triggered by immunopathogenic CD8⁺ T cells [50]. Another ISG, the Ifi27l2a gene, is differentially expressed on neurons by enhancing cell death during WNV infection. As a result, Ifi27l2a protects mice from WNV-induced mortality and prevents virus dissemination in the hindbrain and spinal cord, possibly by regulating the cell death of neurons [51].

#### 3.2.3. Adaptive Immune Responses

CD8^+^ T cells utilize various cytolytic mechanisms to limit WNV infection in the CNS. For example, Fas is strongly upregulated on WNV-infected neurons. CD8^+^ T cells utilize FasL effector mechanisms to restrict WNV infection to Fas-expressing neurons [52]. Likewise, TRAIL is produced by CD8^+^ T cells and contributes to disease resolution by helping to clear WNV infection from neurons in the brain [53]. CD8^+^ T cells also contribute to neuronal pathology during both acute and chronic WNV infection. For example, during a high dose of acute WNV infection, CD8^+^ T cells trigger increased neuron damage [54]. During the recovery stage from acute WNV infection, CNS CD8^+^ T cells or IFN-γ signaling promote microglia-mediated synaptic elimination and, thus, enhance neuronal apoptosis [55].

#### 3.2.4. Ubiquitination Proteins

Accumulation of ubiquitinated abnormal proteins in cells are the outcome of cellular stresses. The accumulation of ubiquitinated proteins in WNV-infected mouse neuroblastoma Neuro-2a cells was reported to be associated with apoptosis in these cells [56]. The underlying mechanisms of ubiquitination-mediated cell death are not well understood. Pellino (Peli)-1, an E3 ubiquitin ligase, is expressed on many cell types and is enriched in the CNS tissues [57]. Peli1 also modulates necroptosis and apoptosis [58]. In a recent report [59], we demonstrated that Peli1 promotes lethal viral encephalitis by facilitating WNV replication in neurons and microglia and induction of neuroinflammation. It is likely that Peli1 contributes to neuron death directly by promoting WNV replication and indirectly by induction of neuroinflammation.

#### 3.2.5. Other Host Factors

CCCTC-binding factor (CTCF), a DNA-binding zinc-finger protein, is involved in antiapoptotic activities presumably via the repression of transcription of proapoptotic genes, such as Bax [61]. The antiapoptotic functions of the epidermal growth factor receptor (EGFR)-coamplified (ECOP) are correlated with enhanced NF-κB activity [62]. CTCF and ECOP genes were the targets of Hs_154, one of the miRNA- induced in WNV-infected neuronal cells or CNS tissues [60]. The miRNA Hs_154 expression could direct the repression of both of these two genes in WNV-induced cell death. Thus, WNV may regulate apoptotic responses in neurons via the induction of miRNA.

## 4. Targeting Cell Death Factors for Treatment of WNV Infection

Prevention of neuronal death can be achieved directly by blocking the apoptosis pathways or indirectly by inhibition of WNV replication. For example, IFN-induced viperin can act as an inhibitor of flaviviruses replication in the brain with a region-specific role in the olfactory bulb and cerebrum. In particular, viperin can efficiently inhibit WNV infection in primary cortical neurons which, which in turn decreases neuronal death [63]. Minocycline, one of the tetracycline-derivatives, is a clinically approved drug that shows high penetration across the blood–brain barrier. Minocycline has multiple effects on treatment of WNV infection. First, it can inhibit WNV-induced apoptosis by blocking caspase-3 activation and PARP cleavage in primary human neuronal cells [64]. Next, c-Jun N-terminal kinase (JNK) signaling has a pro-apoptotic role in neurons [65]. The anti-apoptotic effects of minocycline on neurons were reported to be mediated by both its direct suppression on WNV replication and blockage of the virus-induced activation of JNK and its target c-jun [65]. The limitations of targeting cell death in reversing WNV disease, include the timing, infection severity, and treatment dosage. For example, timing of such treatments is very critical—if too late, the damage cannot be prevented or reversed [66].

## 5. Conclusions

Neuronal loss is a hallmark of WNV-induced meningitis and encephalitis. In the CNS, WNV triggers cell death preferentially in neurons, predominantly via apoptosis following the activation of caspase-9 and caspase-3 pathways and induction of the UPR pathways and ERAD-associated genes. Animal studies suggest that WNV proteins, including the capsid and/or the NS proteins, are involved in mediating apoptosis. Cell death associated host factors, inflammatory cytokines and ISGs, and ubiquitination also contribute to the induction of neuronal death. Results from the animal studies have provided important insights into WNV neuropathogenesis. In addition, targeting the factors that contribute to neuronal death will help to develop new strategies to prevent and treat WNV- induced encephalitis.

## Figures and Tables

**Table 1:** Viral or host factors involved in West Nile virus (WNV)-induced neuronal death.

Viral or Host Factors	Effects on Apoptosis	References
WNV capsid protein	Pro or anti-apoptotic	[22,25,33,34,35,36]
WNV NS3, NS2A protein	Pro-apoptotic	[37,38,39,40]
Caspase 3 or 9	Pro-apoptotic	[25,41]
ER stress, UPR, ERAD	Pro or anti- apoptotic	[37,42,43]
Proinflammatory cytokines	Pro or anti-apoptotic	[44,45,46,47,48,49]
ISGs	Pro-apoptotic	[50,51]
Adaptive immune responses	Pro-apoptotic	[52,53,54,55]
Ubiquitination proteins	Pro-apoptotic	[56,57,58,59]
Others (miRNA Hs_154)	Pro-apoptotic	[60]
